# Distribution of Microaneurysms and Hemorrhages in Accordance with the Grading of Diabetic Retinopathy in Type Diabetes Patients

**DOI:** 10.3390/diagnostics14141547

**Published:** 2024-07-17

**Authors:** Pedro Romero-Aroca, Eugeni Garcia-Curto, Jordi Pascual-Fontanilles, Aida Valls, Antonio Moreno, Marc Baget-Bernaldiz

**Affiliations:** 1 Ophthalmology Service, Hospital Universitario Sant Joan, Universitat Rovira & Virgili, Institut de Investigacio Sanitaria Pere Virgili (IISPV), 43204 Reus, Spain; eugenigorg@gmail.com (E.G.-C.); mbaget@gmail.com (M.B.-B.); 2 ITAKA Research Group, Department of Computer Science and Mathematics, Universitat Rovira & Virgili, Institut d’Investigacions Sanitaries Pere Virgili (IISPV), 43007 Tarragona, Spain; jordi.pascual@urv.cat (J.P.-F.); aida.valls@urv.cat (A.V.); antonio.moreno@urv.cat (A.M.)

**Keywords:** diabetic retinopathy, severe diabetic retinopathy, microaneurysms, retinal hemorrhages, artificial intelligence

## Abstract

(1) Underlying Diabetic Retinopathy (DR) is the primary cause of poor vision in young adults. There are automatic image reading systems that can aid screening for DR. (2) Methods: Using our automatic reading system we have counted the number of microaneurysms and hemorrhages in the four quadrants of the ETDRS grid and evaluated the differences between them according to the type of DR. The study was carried out using data from two different databases, MESSIDOR and MIRADATASET. (3) Results: The majority of microaneurysms and hemorrhages are found in the temporal and inferior quadrants of the ETDRS grid. Differences are significant with respect to the other two quadrants at *p* < 0.001. Differences between the type of DR show that severe-DR has a greater number of microaneurysms and hemorrhages in the temporal and inferior quadrant, being significant at *p* < 0.001. (4) Conclusions: The count of microaneurysms and hemorrhages is higher in the temporal and inferior quadrants in all types of DR, and those differences are more important in the case of severe-DR.

## 1. Introduction

Diabetes mellitus (DM) currently affects 463 million patients worldwide, a number that is expected to increase to as many as 700 million by 2045, according to data from the International Diabetes Federation (IDF) [1,2]. In Spain, diabetes studies [3,4] report that DM affects 14% of the adult population, with type 2 diabetes (T2DM) being the most prevalent, representing 90% (5.3 million) of the cases.

Poorly controlled DM can accelerate complications and cause premature death. It can affect the cardiac and cerebral vascular system, kidneys and vision [5]. Early multifactorial treatments can delay the appearance of complications and thus improve quality of life and life expectancy. Regarding ocular involvement, the most serious complication of DM is diabetic retinopathy (DR), a common cause of blindness in Europe [6]. The duration of the disease, the type of diabetes treatment and the degree of metabolic control are determining factors in its development.

The initial lesion that defines the presence of DR is the presence of microaneurysms; these are a consequence of the weakness of the vascular wall of the capillaries in the venular zone due to the metabolic disorder caused by DM. Likewise, hemorrhages secondary to the rupture of the weakened capillary wall can be observed, and if they are located in the superficial layers of the retina they adopt the shape of a flame, and if they are located in the deep layers they adopt the rounded shape. Both types of hemorrhages differ, and those caused by microaneurysms, in addition to their larger size, are a darker red color.

Screening for DR is essential if we are to avoid an increase in cases of poor vision and blindness in the diabetes population, and is proven to be a cost-effective service [7]. Screening is carried out through evaluation of non-mydriatic retinography taken annually or biannually as recommended by various scientific societies [8,9].

To aid screening, automatic image reading systems have recently been adopted using deep learning techniques, which allow us to determine what stage of DR the patient is at [10]. These systems allow us to count the number of lesions that appear in the retina and to know whether there are microaneurysms or hemorrhages present [11].

The aim of the present study was to determine, by counting microaneurysms and hemorrhages, differences that exist between the various types of non-proliferative DR, that is, whether mild-DR, moderate-DR or severe-DR is observed. To accomplish this, we used a retinal image reading algorithm that had been built by our own research team [12].

## 2. Materials and Methods

### 2.1. Setting

Hospital Univesitario Sant Joan de Reus and Universitat Rovira & Virgili, Tarragona, Spain.

### 2.2. Building the Algorithm

To achieve the main proposed objective, we developed an automatic image reading algorithm able to detect separately microaneurysms, hemorrhages and hard exudates, and which allows us to count each one of them. We describe the construction of the algorithm below. It is an advanced model designed to achieve accurate lesion segmentation from fundus images using deep learning methods. The initial build was carried out using 27 IDRID images [13]. Training, validation and testing of the algorithm was followed by further training using 516 images of patients from our service, different from those of MIRADATASET [14], and finally testing using 1200 images from MESSIDOR [15]. 

The algorithm first detects the papilla to know whether we are working on a left or right eye. The papilla is detected thanks to another model specifically trained to detect the optic disc in the image. Then it detects the microaneurysms and hemorrhages to differentiate between lesions; a specific training was conducted for each one, where they were marked (segmented) separately and verified by a professional ophthalmologist to ensure accurate distinction.

**Figure 1:**
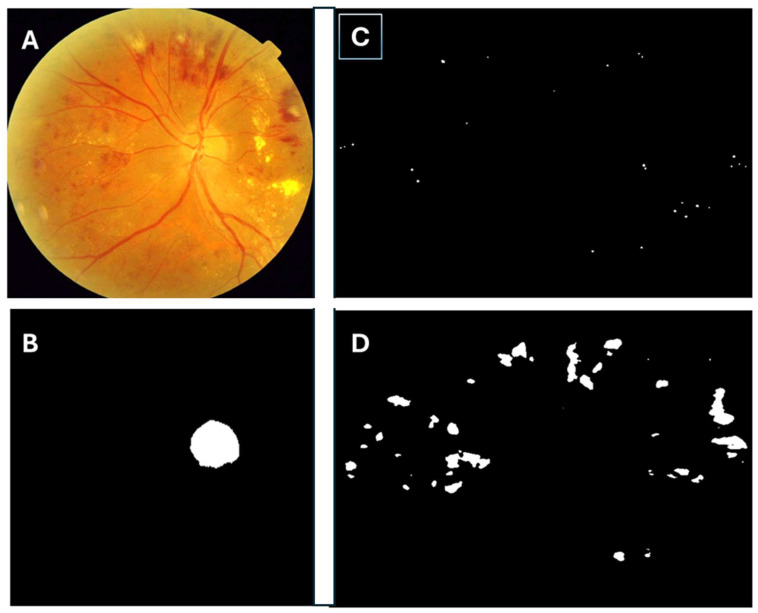
(**A**) Analyzed retinography, (**B**) detection of the papilla, (**C**) detection of the microaneurysms, (**D**) detection of hemorrhages. These figure corresponds to the analysis using the algorithm of the retina image or retinography (**A**). In image (**B**), it is observed how the papilla is isolated to know which eye it is, whether it is the right or the left, in image (**C**), it is observed how the microaneurysms corresponding to retinography (**A**) are isolated, and in image (**D**), the hemorrhages isolated from retinography (**A**) are observed.

The system allows automatic counting of the number of microaneurysms and hemorrhages in the retinography that we present (Figure 1).

### 2.3. Aim

This study presents the results of applying our new lecture algorithm to the MESSIDOR database and to a sample of patients from our own database MIRADATASET. The objective was to determine the differences in the count of microaneurysms and hemorrhages across the quadrants of the ETDRS grid, according to different levels of DR.

### 2.4. Design

Two image databases were used, the MESSIDOR public database [13], which has 1200 retinal images of diabetic patients, and the MIRADATASET database [14], developed by the Ophthalmology Research group of the Institut d’Investigacions Saniteries Pere Virgili (IISPV), which has 40,692 retinal images of patients with DM. From the MESSIDOR database we used all 1200 images and from the MIRSADATASET database we used another 1200 images randomly selected. The distribution of images according to severity is shown in Table 1.

### 2.5. Inclusion and Exclusion Criteria

- Retinography of T2DM patients from MESSIDOR and MIRADATASET were included.
- Retinography of other datasets and with low quality were excluded.

### 2.6. Sample Size

From the two databases in this study we have used the following: from MESSIDOR, all the images shown in Table 1, and from MIRADATASET, we have used a sample of 300 images randomly selected from each of the groups observed in Table 1.

### 2.7. Methods

**Figure 2:**
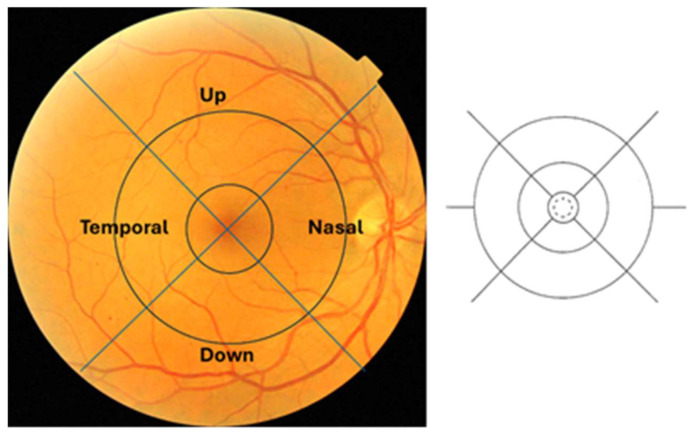
On the (**left**), the modified ETDRS grid. On the (**right**), the grid of the original ETDRS.

The retinography from the two databases have been evaluated taking into account that they were 45° centered on the macula. To count the lesions, the ETDRS grid was modified [16,17] by extending the four quadrants to the periphery of the 45° retinography, dividing it into four equal parts, centering the grid at the level of the fovea (Figure 2). Using the new grid, the number of microaneurysms and hemorrhages were counted automatically in each of the four quadrants. Two ophthalmologists, co-authors of this study, subsequently verified manually that the number counted was correct.

### 2.8. Classification of Diabetic Retinopathy

The following MESSIDOR database classification [14] was used in the study:

1. No DR, level 0: (μA = 0) AND (H = 0)
2. Mild-DR, level 1: (0 < μA ≤ 5) AND (H = 0)
3. Moderate-DR, level 2: ((5 < μA < 15) OR (0 < H < 5)) AND (NV = 0)
4. Severe-DR, level 3: (μA ≥ 15) OR (H ≥ 5) OR (NV = 1)

Where μA = number of microaneurysms, H = number of hemorrhages, NV = 1: neovascularization and NV = 0: no neovascularization.

Although this classification does include diabetic macular edema, we did not use it in the present study as it was not our objective to determine the presence or absence of macular edema.

### 2.9. Ethics and Consent

The study was carried out with the approval of the local ethics committee, the Institutional Review Ethics Committee (CEIM (Comite de Etica en Investigaciones Medicas)) of Institut d’Investigacio Sanitaria Pere Virgili (IISPV), Tarragona, Spain, approval code RetinaReadRisk, protocol version 1., 10 March 2022, Reference number CEIM: 071/2022.

### 2.10. Statistical Methods

Data were analyzed using SPSS, version 22.0 (IBM^®^ Statistics, Chicago, IL, USA). Descriptive statistical analysis of quantitative data was made by determining the mean and standard deviation. For qualitative data we used the analysis of frequency and percentage in each category. For all parameters, descriptive statistics (including the number of values, mean, standard deviation (SD) and standard error of the mean (SEM)) were calculated for each microvascular lesion (microaneurysms, hemorrhages and hard exudates) for each DR grade level. The statistical study of the quantitative variables was carried out by applying the Student’s *t* test to compare the means between two groups and the ANOVA test when there were more than two groups. The differences were considered positive at *p* ≤ 0.05. We determined the differences in the means of the microaneurysm and hemorrhage counts of the four quadrants of the expanded ETDRS grid and the means of the types of DR in each quadrant. Studies were carried out separately on the images from the MESSIDOR database and our MIRADATASET database.

## 3. Results

### 3.1. Analysis of the Two Databases MESSIDOR and MIRADATASET

MESSIDOR. The 1200 fundus color-numerical images of the posterior pole were acquired by three ophthalmology departments using a color video 3CCD camera Topcon TRC NW6 non-mydriatic retinal camera with a 45 degree field of view. The images were captured using 8 bits per color plane at 1440 × 960, 2240 × 1488 or 2304 × 1536 pixels. A total of 800 images were acquired with pupil dilation (one drop of Tropicamide at 0.5%) and 400 without dilation. The images were evaluated by the three ophthalmologists based on 3 sets of 400 images stored in TIFF for reading.

MIRADATASET. The 40,692 images were acquired from a TOPCON TRC NW6 non-mydriatic retinal camera with a 45 degree field of view. Images were captured using a color retinography of 1440 × 960, 2240 × 1488 or 2304 × 1536 pixels. Three retinographies were obtained using the JOSLIN system, one centered on the macula, another on the nasal side of the papilla and a third on the superior temporal. The images were stored in DICOM and were then classified by two pairs of ophthalmologists in our service using the double-blind system. In any cases of doubt, the two pairs came to a consensus. In addition to the retinography, OCT examinations were carried out to rule out the presence of diabetic macular edema, and OCTA and fluorescein angiography of the retinal periphery were carried out to correctly classify patients as moderate-DR and severe-DR. The final classification was made according to the MESSIDOR system and retinographies centered on the macula were chosen to build the database.

### 3.2. MESSIDOR Database Study

#### 3.2.1. Microaneurysm Count

Table 2 shows that the majority of microaneurysms are concentrated in the temporal and inferior areas. This happens at all stages of DR and mostly at severe-DR.

#### 3.2.2. Hemorrhages Count

Table 2 shows that with respect to hemorrhages, it is in the severe-DR form where the greatest difference between areas is manifested, especially in the temporal area and secondly in the inferior area, which is where the greatest number of hemorrhages are concentrated. Regarding the mild-DR and moderate-DR forms, the greatest difference is found in the temporal area compared to the other three, where most of the hemorrhages are concentrated.

### 3.3. Analysis of the MIRADATASET Database

#### 3.3.1. Microaneurysm Count

Regarding the microaneurysm count, Table 3 shows that a greater number are found in the temporal (7.51 ± 7.72) and inferior areas (7.46 ± 7.67), and the difference between these two areas is minimal.

#### 3.3.2. Hemorrhage Count

Regarding the hemorrhage count, Table 3 shows a similar result. There is a greater concentration in the temporal (3.13 ± 4.81) and inferior (3.19 ± 5.30) areas, and the differences between them are minimal.

### 3.4. Results at Central Ring

In the 1500 micron diameter of the central ring of the ETDRS, corresponding to the fovea, no microaneurysms or hemorrhages were found, consistent with the lack of a vascular network in this area that contains the central avascular zone.

### 3.5. Statistical Analysis

Applying ANOVA to the MESSIDOR database shows that the differences between the four quadrants for each type of DR are significant at *p* < 0.001, and are similarly significant if we evaluate the mean differences for each quadrant by type of DR at *p* < 0.001. Applying ANOVA to the MIRADATASET database shows that the differences between the four quadrants for each type of DR are significant at *p* < 0.001 values, and are similarly significant if we evaluate the mean differences for each quadrant by type of DR with *p* < 0.001. 

Applying the Student’s test to the MESSIDOR database for differences between groups, we only found significant differences for severe-DR. Table 4 shows statistical analysis, differences between temporal and nasal, upper and inferior quadrants were significant for microaneurysms and hemorrhages. 

Applying Student’s test to observe differences between groups in the MIRADATASET database, significant differences were observed only for severe-DR. Table 4 shows differences between temporal and nasal, upper and inferior quadrants. Differences were significant for temporal versus nasal and upper quadrants, but not for temporal versus inferior quadrant.

In summary, ANOVA demonstrated differences in microaneurysm and hemorrhage distribution between all quadrants, but the application of Student’s *t* test demonstrated that these differences were only for severe-DR. Furthermore, we observed that in severe-DR with Student’s *t*, in the MESSIDOR database, microaneurysms and hemorrhages were more concentrated in the temporal sector and the differences were significant with respect to the other three quadrants, but in the MIRADATASET database, microaneurysms and hemorrhages were more concentrated both in the temporal and in the inferior sector, the differences with the other two quadrants being significant.

## 4. Discussion

The automatic detection of DR lesions has become necessary given the exponential increase in DM in the population. Among some 800 million patients predicted that, by the year 2045, around 20 to 30% of it will have some form of DR, diabetic retinopathy, with the concomitant risk of poor vision or blindness [18,19]. To avoid this, there needs to be some form of screening of all patients with DM. Given the number of patients affected, various professionals, general practitioners, pediatricians and endocrinologists among them, together with ophthalmologists, need to be part of the screening programs. The large number of affected patients will create the need for diagnostic aids involving automatic image reading systems built using artificial intelligence techniques.

In the present study, the results of the algorithm that we have developed not only detects the presence or not of DR but also counts the number of lesions in the retina (microaneurysms, hemorrhages and hard exudates) across the four quadrants of the ETDRS grid extending to the entire surface of the 45° periphery of the retinography. The results show that the distribution of microaneurysms and hemorrhages is not random across the retina but is distributed irregularly depending on their location in the extended ETDRS grid. It is very interesting that both microaneurysms and hemorrhages concentrate in greater numbers in the temporal and inferior quadrants, especially in severe-DR. A statistically significant distribution of microaneurysms and hemorrhages concentrated in the temporal quadrant, and to a lesser extent in the inferior quadrant, might therefore be a way of allowing us to begin classifying DR in patients undergoing screening and to refer those with a predominance of lesions on the temporal side, who will be at risk of severe-DR, to an ophthalmologist. The majority of other similar systems that have been developed are of the Deep Learning type [20]. This type of learning, called Supervised type, requires prior marking of the lesions on the retinography and their labelling by type of DR before being applied for training. Initially, the network makes many errors in the classification of the retinography, but are then computed to improve the classifier function of the neural network. Once the neural network reads the set of retinography repeatedly (iterations), the neurons adjust their parameters (called ‘weights’) until they become a good DR classifier. The automatic reading systems currently on the market use this deep learning technique but do not allow the counting of lesions individually, but instead classify DR globally according to the distribution and number of lesions in the retinography without giving the number of microaneurysms or hemorrhages [21,22].

In the literature there are few studies that focus on counting the number of retinal lesions. The most notable are those of the Cunha Vaz group [23,24], who have developed an algorithm that detects the turnover of microaneurysms in the macular area and from there determines the risk of progression of DR. Likewise, this team has developed the hypothesis of the existence of three phenotypes of diabetes patients [24,25,26]. Phenotype A are those patients who develop DR over the years of DM evolution, that is to say, progression towards the appearance of DR would be slow, Phenotype B are those who are more susceptible to a breakdown in the blood-retinal barrier and the appearance of wet or edematous forms of DR, and Phenotype C are those linked to obstruction due to microthrombosis or capillary occlusion that would give rise to ischemic forms of DR. However, the Cunha Vaz group’s study only focused on the macular area, unlike the present study in which we have identified lesions across the entire retinography.

Another study, Munuera et al. [27] indicated that microaneurysms and hemorrhages are distributed in a similar way to what we have described, but due to the small sample of only 94 patients and the fact that they used the lesion classification system in the cross-shaped retina centered on the fovea and not the ETDRS grid makes it difficult to compare our results. That study did, however, identify a distribution that tends to locate the lesions more in the temporal quadrants.

Regarding the cause of the highest concentration of lesions (microaneurysms and hemorrhages in the temporal and inferior quadrants), it is unknown. A possible explanation could be embryological; since in the fetus the inferior fissure is the last one to close, the vascularization of the inferior retina could be affected by closure defects and with respect to the temporal retina perhaps given that the growth of the vascularization. The retina grows in the shape of circles from the optic disc, the temporal retina is the last to form and defects can be generated.

Another possible explanation would be metabolic through the protease activity of caspase 1, studied by Tien et al. [28], who demonstrated differences in the development of microaneurysms, these being more abundant in the temporal sector versus the nasal sector. According to Tien et al., the apoptosis of retinal capillary cells begins early in DM and probably contributes to capillary obliteration; these authors suggest that caspases, which are proteolytic enzymes closely involved in cell apoptosis, especially caspase-1, which has been increased in DM and is distributed in a non-uniform manner so that the temporal retina has greater activation of caspase-1, play an important role in the development of DR and in the different distribution of microaneurysms.

One strength of the present study is that we have carried it out using two different databases (MESSIDOR and MIRADATASET). Another important strength is that since the algorithm is built and tested on retinographies from a database of patients from our own population (MIRADATASET), applying the algorithm to it is always safer than applying it to different populations that might have retinas with different pigmentation. A weakness of the study is that the MESSIDOR classification that defines the degrees of DR does not coincide with the international classification generally used in clinical practice. Another weakness is that the study has been based on a single 45° retinography centered between the macula and the temporal side of the papilla, rather than on the seven fields of the ETDRS [29] or the three of the Joslin technique [17]. We know that if we use wide-field systems, the severity of DR can change. For example, in Domalpally et al. [30] the peripheral lesions contributed to a higher DR severity in 8% of cases and changed the eye to a proliferative DR level in 2%. Finally, the paucity of clinical data on the patients made it difficult to associate the lesions that we observed with good or bad metabolic control of the patients, or to link them to other demographic data, such as age, sex or race.

## 5. Conclusions

The algorithm detects microaneurysms and hemorrhages well. Counting them suggests that the temporal and inferior quadrants, in that order, are where the lesions are preferentially located, and they increase in number with the severity of the DR. More studies are required to confirm that by evaluating the temporal and inferior quadrants, we were able to classify DR. 

## Figures and Tables

**Table 1:** Number of images in each used database.

Classification	MESSIDOR	MIRADATASET
No DR	609	37,218
Mild-DR	182	715
Moderate-DR	162	1290
Severe-DR	261	1469
Total	1200	40,692

**Table 2:** Results of injury counting in the MESSIDOR database.

Microaneurysms	**Type of DR**	**UP**	**TEMPORAL**	**NASAL**	**DOWN**	**ANOVA**
	Mild-DR	1.25 ± 0.71	1.01 ± 1.08	0.38 ± 0.62	0.86 ± 0.92	<0.001
	Moderate-DR	1.64 ± 0.92	1.50 ± 2.07	0.65 ± 1.13	1.01 ± 1.27	<0.001
	Severe-DR	2.47 ± 2.80	5.50 ± 6.52	1.86 ± 2.22	3.10 ± 3.23	<0.001
ANOVA test	<0.001	<0.001	<0.001	<0.001	<0.001	
Hemorrhages	**Type of DR**	**UP**	**TEMPORAL**	**NASAL**	**DOWN**	
	Mild-DR	1.07 ± 0.27	1.53 ± 0.77	1.12 ± 0.48	1.17 ± 0.64	<0.001
	Moderate-DR	1.26 ± 0.50	1.69 ± 0.98	1.29 ± 0.55	1.31 ± 0.58	<0.001
	Severe-DR	2.08 ± 3.44	4.98 ± 6.55	1.96 ± 2.66	2.18 ± 3.22	<0.001
ANOVA test	<0.001	<0.001	<0.001	<0.001	<0.001	

**Table 3:** Result of the injury count in the MIRADATASET database.

Microaneurysms	**Type of DR**	**UP**	**TEMPORAL**	**NASAL**	**DOWN**	**ANOVA Test**
	Mild-DR	1.65 ± 1.10	1.18 ± 1.51	1.86 ± 1.16	1.01 ± 1.25	<0.001
	Moderate-DR	1.04 ± 1.33	2.23 ± 2.85	1.47 ± 1.72	1.76 ± 2.05	<0.001
	Severe-DR	4.72 ± 4.19	7.51 ± 7.72	5.16 ± 4.78	7.46 ± 7.67	<0.001
ANOVA test	<0.001	<0.001	<0.001	<0.001	<0.001	
Hemorrhages	**Type of DR**	**UP**	**TEMPORAL**	**NASAL**	**DOWN**	**ANOVA test**
	Mild-DR	1.34 ± 0.76	1.39 ± 0.80	1.24 ± 0.56	1.29 ± 0.64	<0.001
	Moderate-DR	1.52 ± 0.85	1.76 ± 1.25	1.55 ± 0.88	1.45 ± 0.85	<0.001
	Severe-DR	2.28 ± 3.25	3.13 ± 4.81	2.47 ± 3.79	3.19 ± 5.30	<0.001
ANOVA test	<0.001	<0.001	<0.001	<0.001	<0.001	

**Table 4:** Student’s *t* test analysis.

Database	Lesions	Quadrants	Significance, (t Value)
MESSIDOR	Microaneurysms	Temporal versus nasal	*p* < 0.001, (t = 8.53),
		Temporal versus upper	*p* = 0.02, (t = 6.89),
		Temporal versus inferior	*p* = 0.03, (t = 5.34)
	Hemorrhages	Temporal versus nasal	*p* < 0.001, (t = 7.72)
		Temporal versus upper	*p* = 0.02, (t = 6.33)
		Temporal versus inferior	*p* = 0.02, (t = 6.19).
MIRADATASET	Microaneurysms	Temporal versus nasal	*p* = 0.03, (t = 3.53)
		Temporal versus upper	*p* = 0.02, (t = 4.89)
		Temporal versus inferior	*p* = 0.87, (t = 1.04)
	Hemorrhages	Temporal versus nasal	*p* = 0.03, (t = 2.72),
		Temporal versus upper	*p* = 0.04, (t = 2.33)
		Temporal versus inferior	*p* = 0.91, (t = 1.02).

## Data Availability

The original contributions presented in the study are included in the article, further enquiries can be directed to the corresponding authors.
